# Loneliness and Well-Being in Children and Adolescents during the COVID-19 Pandemic: A Systematic Review

**DOI:** 10.3390/children10020279

**Published:** 2023-01-31

**Authors:** Ann H. Farrell, Irene Vitoroulis, Mollie Eriksson, Tracy Vaillancourt

**Affiliations:** 1 Department of Child and Youth Studies, Brock University, St. Catharines, ON L2S 3A1, Canada; 2 School of Psychology, Faculty of Social Sciences, University of Ottawa, Ottawa, ON K1N 6N5, Canada; 3 College of Health and Science, DePaul University, Chicago, IL 60614, USA; 4 Counselling Psychology, Faculty of Education, University of Ottawa, Ottawa, ON K1N 6N5, Canada

**Keywords:** COVID-19 pandemic, children, adolescents, loneliness, well-being, mental health, social isolation, systematic review, anxiety, depression

## Abstract

Concerns have been raised about the loneliness and well-being of children and adolescents during the COVID-19 pandemic. The extent to which the ongoing pandemic has impacted loneliness and the association between loneliness and well-being is unclear. Therefore, a systematic review of empirical studies on the COVID-19 pandemic was conducted to examine the (1) prevalence of loneliness in children and adolescents, (2) associations between loneliness and indicators of well-being, and (3) moderators of these associations. Five databases (MEDLINE, Embase, PsycInfo, Web of Science, ERIC) were searched from 1 January 2020 to 28 June 2022 and 41 studies met our inclusion criteria (cross-sectional: *n* = 30; longitudinal: *n* = 11; registered on PROSPERO: CRD42022337252). Cross-sectional prevalence rates of pandemic loneliness varied, with some finding that over half of children and adolescents experienced at least moderate levels of loneliness. Longitudinal results reflected significant mean increases in loneliness compared to pre-pandemic levels. Cross-sectional results indicated that higher levels of loneliness were significantly associated with poorer well-being, including higher depression symptoms, anxiety symptoms, gaming addiction, and sleep problems. Longitudinal associations between loneliness and well-being were more complex than cross-sectional associations, varying by assessment timing and factors in the statistical analyses. There was limited diversity in study designs and samples, preventing a thorough examination of moderating characteristics. Findings highlight a broader challenge with child and adolescent well-being that predates the pandemic and the need for future research to examine underrepresented populations across multiple timepoints.

## 1. Introduction

The social relationships of children and adolescents were significantly disrupted because of the COVID-19 pandemic mitigation measures such as lockdowns and physical distancing practices. For example, approximately 90% of the world’s children and adolescents were impacted by school closures [1]. These mitigation measures, although important in reducing the spread of the COVID-19 virus, prevented children and adolescents from seeing their friends, peers, and broader social networks (e.g., teachers, coaches, teammates) in person. Social relationships are increasingly important across childhood and adolescence for healthy identity formation and mental well-being [2]. Social isolation, defined as the lack of social contact [3] can often lead to feelings of loneliness, defined as the discrepancy between desired and perceived relationships [4]. Loneliness is a significant problem because humans are evolutionarily “wired” to belong to stable and secure social relationships [5]. When perceived social isolation thwarts this fundamental need, loneliness can ensue and result in poorer mental well-being. Due to the range of different measures used by researchers, in this review, well-being will be used subsequently as an overarching term that encompasses indicators of global well-being and/or mental health and specific indicators such as depressive symptoms, anxiety symptoms, internalizing symptoms, and externalizing symptoms [6]. Given these associations, the increases in loneliness and poor well-being have been concerns during the pandemic. As the pandemic reaches its third-year anniversary, it is important to review the evidence comprehensively and systematically on the impact of the pandemic on loneliness and well-being to understand the current and long-term implications for children and adolescents.

### 1.1. Loneliness and Well-Being before the Pandemic

Loneliness is not new a problem for children and adolescents. In one study from before the pandemic, time trends of eight-year-olds’ loneliness across 24 years showed that up to 20% of children consistently reported feeling lonely [7]. In a recent meta-analysis of pre-pandemic studies (i.e., studies conducted prior to the pandemic), the prevalence rates of loneliness among adolescents between ages 12 and 17 across 76 countries ranged from 9.2% to 14.4% depending on the geographic region [8]. Data from the Organisation for Economic Co-operation and Development, comprising nationally representative samples of 15- to 16-year-old adolescents across 37 countries, showed that mean levels of feelings of loneliness experienced at school (weighted by population) increased between 2000 (*M* = 1.83, *SD* = 0.46) to 2018 (*M* = 2.02, *SD* = 0.55; *d* = 0.36), with most of the increases occurring between 2012 (*M* = 1.84, *SD* = 0.48) to 2018 (*d* = 0.35; [9]).

In pre-pandemic studies, researchers have consistently shown that loneliness in children and adolescents confers a risk for poorer well-being. For example, loneliness was longitudinally related with depression symptoms from childhood to adolescence [10] and loneliness longitudinally co-developed with depression and social anxiety symptoms across adolescence [11]. Loneliness at age eight was also concurrently associated with psychiatric symptoms such as conduct problems, hyperactivity, and emotional problems [7]. Considering that over 50% of mental health problems develop during childhood and adolescence [12], with the mean age of onset of mental disorders being 14.5 [13], poorer well-being that results from loneliness may have long-term implications for health and wellness in adulthood. Therefore, it is important to understand how the pandemic impacted loneliness rates in children and adolescents and how loneliness affected their well-being. What is not clear is the extent to which the pandemic has exacerbated pre-pandemic loneliness or contributed to new experiences of loneliness. It is also not clear how loneliness in the context of the pandemic contributed to well-being in children and adolescents.

### 1.2. Loneliness and Well-Being during the Pandemic

At the beginning of the pandemic, there was a push to rapidly disseminate cross-sectional studies to better understand its impact on well-being. Since the initial wave, an increasing number of new peer-reviewed longitudinal studies capturing follow-up assessments and subsequent waves of the pandemic have emerged. As a result, several systematic reviews and meta-analyses synthesizing these findings have been published. Systematic reviews involve explicit and methodical approaches to search, identify, collate, and synthesize results from studies that address a particular research question [14]. When enough statistical effect size estimates and their variances are evident, they can be quantitatively summarized in what is known as a meta-analysis [14]. However, only some of these systematic reviews and meta-analyses included the prevalence of child and adolescent loneliness (e.g., [15]) and even fewer have focused on loneliness with the well-being of children and adolescents (e.g., [16]).

What is currently known about the prevalence of loneliness during the pandemic based on systematic reviews and meta-analyses has been largely limited to adult samples. For example, Buecker and Horstman [17] systematically reviewed 53 studies on the prevalence and correlates of adulthood loneliness during the early phase of the pandemic and found that most studies were cross-sectional. The few longitudinal studies found increases in loneliness compared to pre-pandemic measurements assessed months to years before the pandemic. However, stability or decreases were found when pre-pandemic measurements were a few weeks or days before the pandemic. More recently, Ernst et al. [15] examined the prevalence rates of loneliness in a systematic review with meta-analysis of longitudinal studies. Of the 34 studies included in the systematic review, only two were on adolescent samples and the remaining studies were on adult samples. Loneliness scores (19 studies) and prevalence rates (eight studies) revealed increases in pandemic scores and rates relative to pre-pandemic scores and rates, with a small effect size.

The impact of the pandemic on child and adolescent well-being has been another focus of several systematic reviews [18,19] and meta-analyses [20,21]. The reviews that were conducted earlier in the pandemic primarily included cross-sectional studies. For example, several systematic reviews on children and adolescents found that the pandemic negatively impacted depression and anxiety symptoms (ages 0 to 18 [22,23]), sadness, loneliness, and hyperactivity (kindergarten to high school [24]). Meta-analytic findings of children and adolescents between ages 0 and 18 have shown that depression and anxiety symptoms have increased by approximately double that of pre-pandemic estimates with 1 in 4 experiencing clinically elevated depression symptoms and 1 in 5 experiencing clinically elevated anxiety symptoms [21]. These estimates were higher in studies that were conducted later in the pandemic. Several systematic reviews also found that certain child and adolescent populations were at risk for experiencing poorer well-being outcomes, including older adolescents, girls, neurodivergent populations, and/or chronic physical conditions [23], and populations with psychiatric or developmental disorders (e.g., obesity, lung disease, attention deficit hyperactivity disorder (ADHD), obsessive compulsive disorder; ages 4 to 19 [25]). Most of these studies were cross-sectional, thus precluding evidence about intraindividual change in well-being.

Another limitation of these reviews is that they did not examine the impact of the pandemic *on the association* between loneliness and well-being in children and adolescents. However, this information is needed because pre-pandemic systematic reviews and meta-analyses have found loneliness robustly impacts well-being (e.g., depression symptoms [26]). Given the unprecedented nature of this global pandemic, several systematic reviews on the association between child and adolescent loneliness and mental health were conducted during the pandemic but *were based on empirical studies conducted before the pandemic*. In other words, these reviews included empirical studies from before the pandemic with the aim to help inform researchers, practitioners, and policy makers about *what to expect during the pandemic*. For example, Loades et al. [16] and Hards et al. [27] conducted rapid systematic reviews on loneliness and mental health on samples with a mean age of 21 or younger in populations without and with pre-existing mental health conditions, respectively. In both reviews, significant associations were found between loneliness and mental health difficulties including anxiety and depression symptoms, with most studies being cross-sectional in nature. As an increasing number of peer-reviewed publications are continuing to emerge into the third year of the pandemic, an updated systematic review is needed.

### 1.3. Current Study

Our goal was to conduct a systematic review of empirical studies from around the world to examine: (1) the prevalence of loneliness in children and adolescents throughout the pandemic, (2) the associations between loneliness and indicators of well-being throughout the pandemic, and (3) any moderators of the association between loneliness and well-being (e.g., study design, study timing, underrepresented youth populations). We predicted that the prevalence of loneliness would increase among children and adolescents during the pandemic and that higher loneliness would be associated with poorer well-being. Studies conducted later in the pandemic that assess follow-up time points and capture subsequent pandemic waves were expected to demonstrate stronger associations between loneliness and poorer well-being. We also predicted that underrepresented youth populations (e.g., clinical populations, minority race/ethnicity and gender/gender identity groups, immigrants and refugees, and geographic regions) would generally demonstrate higher levels of loneliness and poorer well-being.

## 2. Materials and Methods

### 2.1. Search Strategy and Inclusion Criteria

We followed the Preferred Reporting Items for Systematic Reviews and Meta-Analyses (PRISMA) reporting guidelines [14] for our data reporting and analysis. This systematic review was registered on PROSPERO (CRD42022337252). Searches were conducted on the following databases (platforms in parentheses): MEDLINE (Ovid), Embase (Ovid), PsycInfo (PsycNet), Web of Science (Full Core Collection), and ERIC (ProQuest). The final search was conducted on 28 June 2022, with the time restriction placed from 1 January 2020, up to and including the date of the search. The search strategy was based on four main themes: (1) the COVID-19 pandemic, (2) child or adolescent samples (ages 0–20), (3) loneliness or a similar construct (e.g., belonging, social isolation, mattering), and (4) well-being (e.g., global mental health/well-being, depression/anxiety, internalizing/externalizing symptoms, substance use, and others; see Supplemental Materials Table S1 for a sample of the search strategy). Although our maximum sample age initially was 18, we increased this to 20 to include studies on adolescents that met all inclusion criteria except for having a maximum age of 19 or 20. Loneliness and social isolation are distinct constructs, but we included both to be consistent with previous systematic reviews and meta-analyses on child and adolescent loneliness and/or well-being (e.g., [16,27]).

Considering the rapidly growing literature on the impact of the pandemic on children and adolescents, additional inclusion criteria were used to manage the literature. The final inclusion criteria were: (1) peer-reviewed published empirical and original publications, (2) quantitative studies, (3) studies in English, (4) studies conducted on the COVID-19 pandemic, (5) samples of children and adolescents between ages 0 and 20, (6) studies including an assessment of loneliness or a similar construct (e.g., belonging, social isolation, mattering), (7) studies including an assessment of well-being or similar construct (e.g., global mental health/well-being, depression/anxiety, internalizing/externalizing symptoms, substance use, and others), and (8) studies examining the association between loneliness and well-being. Theoretical reviews, systematic reviews, meta-analyses, unpublished manuscripts, pre-prints, conference abstracts, theses/dissertations, and qualitative studies were excluded. Some studies had large age ranges that included children and adolescents. Therefore, studies were excluded if they did not present results stratified by age specifically on children or adolescents.

### 2.2. Study Selection

Studies retrieved from databases were imported into Covidence software [28]. Duplicates were automatically removed by Covidence. Once duplicates were removed, two authors (AF and IV) double-coded all titles and abstracts against the inclusion criteria (percentage agreement = 0.89, mean random agreement probability = 0.74, κ = 0.60). Disagreements were resolved through consensus. The remaining studies were divided for full-text screening by another two authors (AF and ME).

## 3. Data Extraction

After full-text screening, the remaining studies were divided between AF, IV, and ME for data extraction and all data extracted were double checked by AF. Disagreements were resolved through consensus. The following data were extracted in Microsoft Excel: author(s), country, publication year, study design (cross-sectional/longitudinal), sample age, sample age category (child: 0–11; adolescent: 12–20), sample type (clinical/community), mean age (standard deviation), percentage boys/male and girls/female (non-binary/other if reported in primary studies), measure of loneliness, measure(s) of well-being, loneliness prevalence (i.e., frequency or mean) if provided, and association(s) between loneliness and indicator(s) of well-being. When multiple analyses and statistics were reported in studies, either the main univariate and/or multivariate results were extracted, or a summary description of the main pattern of results across analyses were extracted. We also recorded any additional study and sample characteristics that could potentially be moderators for the results if provided, such as the timing of data collection (e.g., month/year, before, during, or after school closure) and underrepresented populations (e.g., clinical populations, race/ethnicity, gender identity groups, immigrants and refugees, and geographic region). The impact of social media on loneliness was not examined as a potential moderator. Some studies have found that loneliness was associated with social media use among adolescents as a strategy to cope during the pandemic (e.g., [29]). However, this relation went beyond the scope of our systematic review and was thus not included in our analyses.

### 3.1. Quality Assessment

Nine questions adapted from the National Institute of Health Quality Assessment Tool for Observation Cohort and Cross-Sectional Studies were used for quality assessment [30]. Quality assessment was conducted during the data extraction phase (see section above). Questions included whether: (1) research objectives were clear, (2) study population was clear, (3) participation rate was at least 50%, (4) justification for sample size, (5) a clear, valid, and reliable measure of loneliness, (6) a clear, valid, and reliable measure of well-being, (7) the study was longitudinal, (8) analyses were appropriate, and (9) confounding variables were controlled. Each question was answered as either ‘No’ = 0 or ‘Yes’ = 1 and totaled. If the answer was unclear or not provided, studies were given a ‘No’ = 0. Responses were totaled for a possible score between 0 and 9. Longitudinal studies had one additional question on whether the sample loss was less than 20% after baseline, for a possible total score between 0 and 10 (see Supplemental Materials Figure S1 for quality assessment questions).

### 3.2. Analytic Plan

After extracting data from studies meeting the inclusion criteria, data were first organized in a descriptive table grouped by study design. One table was created for cross-sectional studies and a second table was created for longitudinal studies. Results of the two tables were narratively synthesized based on cross-sectional findings followed by longitudinal findings. Synthesis of findings were organized by (1) prevalence of loneliness if provided, (2) indicator of well-being examined with loneliness, (3) association found between loneliness and well-being (e.g., correlation, odds ratio), and (4) timing of study. Descriptive information on study location and sample type (child/adolescent, community/clinical) were also synthesized narratively. If enough data were available for different study and sample characteristics, we planned to synthesize results by these characteristics as potential moderators (e.g., timing of data collection, age of participants, race/ethnicity, gender, underrepresented populations).

## 4. Results

### 4.1. Study Selection

**Figure 1:**
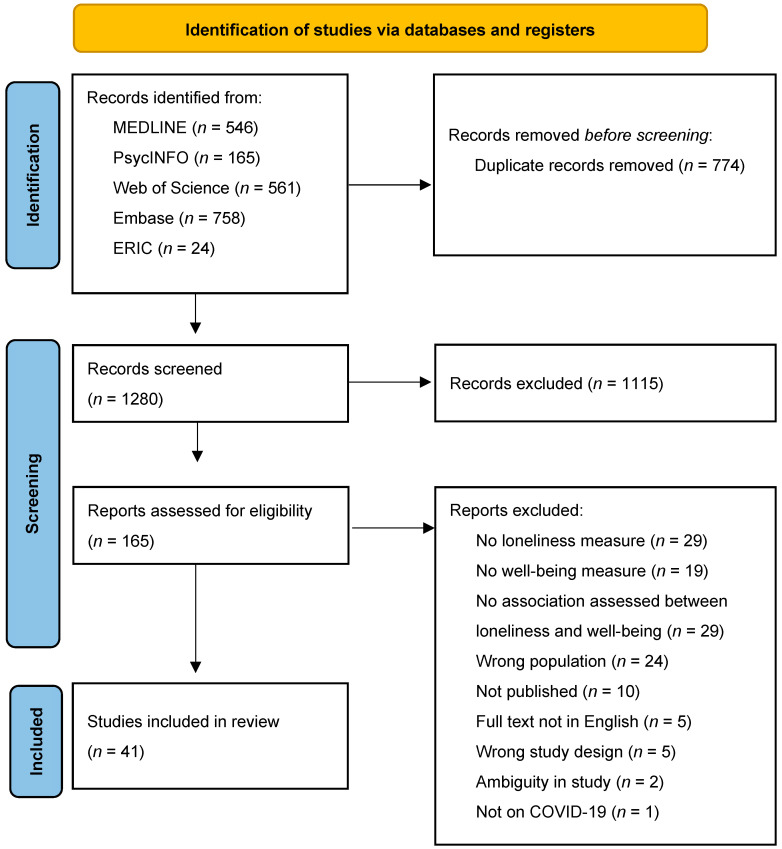
PRISMA Flow Diagram for Systematic Review Study Selection. Note. PRISMA = Preferred Reporting Items for Systematic Reviews and Meta-Analyses. From Page et al. [14].

Our search resulted in 2054 potentially eligible studies for screening (see Figure 1). Duplicates were removed automatically through Covidence and through manual coding by the authors. After removal of duplicates (*n* = 774), 1280 studies were screened for titles and abstracts that met the inclusion criteria. After removing ineligible studies (*n* = 1115), 165 studies remained for full-text screening. This resulted in 41 studies that met the inclusion criteria that were included in the systematic review.

### 4.2. Summary of Studies and Quality Assessment

The summary of cross-sectional studies and longitudinal studies are presented in Table 1 and Table 2, respectively. Of the 41 studies, 30 were cross-sectional and 11 were longitudinal. Most studies were from Europe (*n* = 14), followed by the United States (*n* = 8), Australia (*n* = 6), and China (*n* = 5). The remaining countries included Canada (*n* = 2), Hong Kong (*n* = 2), Brazil (*n* = 2), Israel (*n* = 1), and Chile (*n* = 1). Most of the studies were on adolescent samples (*n* = 27) or both child and adolescent samples (*n* = 11), and 3 studies were on child samples. Most of the studies (*n* = 37) were on community samples and four studies were on both community and clinical samples.

All but four of the cross-sectional studies included self-report measures of loneliness. Several studies used single item self-reports pertaining to loneliness experienced during the pandemic. The four exceptions to self-report measures were Dondi et al. [32], Laslo-Roth et al. [43], Gilsbach et al. [38], and Low and Mounts [47] who used parent-report measures ([38] also included youth reports). All but two longitudinal studies included self-report measures of loneliness. One longitudinal study used a single item of “How often do you feel lonely?” [69] and one study used ecological momentary assessments (EMA) of loneliness three times a day for seven days [66]. The two exceptions from longitudinal studies included a parent-report comprising a single item on perceptions of their child’s loneliness [68,70].

The mean quality assessment for cross-sectional studies (out of 9) was 5.90 (*SD* = 1.12; Min = 4, Max = 8) and for longitudinal studies (out of 10) was 7.18 (*SD* = 1.53; Min = 5, Max = 9). The most common limitations for quality were not providing information on the participation rate of eligible persons or not providing justification of sample size or a power description. Several studies also used measures of loneliness or well-being that were not clearly defined or did not provide information on validity. For example, some studies used a single item referring to loneliness and/or mental health during the pandemic developed for that study (e.g., [38,40]).

## 5. Prevalence of Loneliness

Findings on the prevalence of loneliness were mixed. In cross-sectional studies, the prevalence rates of feeling lonely varied. Fogarty et al. [37] found 38.7% of adolescents reported feeling moderately to extremely lonely and similarly Wang et al. [57] found 33.9% of Chinese adolescents reported feeling lonely. Jones et al. [40] found among adolescents in the United States that 53.4% felt that they did not feel close to people at their school. Dondi et al. [32] used reports by parents in Italy and found 67.6% reported their child felt lonely. Fernandes et al. [36] found 8.5% of Brazilian adolescents felt *extremely* isolated, 20.1% felt *very* isolated, 41.4% felt *moderately* isolated, 19.8% felt *slightly* isolated, and 10.2% did *not* feel isolated. Li et al. [44] found among Australian adolescents that 51.4% felt lonely *often*, 30.7% felt lonely *some* of the time, and 17.1% *hardly ever* felt lonely. One longitudinal study by Zuccolo et al. [70] examined frequency counts of how often parents perceived their child or adolescent felt lonely and found similar proportions among people who reported at baseline (June to November 2020) and during follow-up (2021). Loneliness was categorized into never/almost never (baseline group: 33.04%; follow-up group: 32.25%), a few times (baseline group: 53.10%; follow-up group: 52.96%), and often (baseline group: 13.86%; follow-up group 14.79%), meaning that in total over 66% of children and adolescents felt lonely at least a few times or more. The remaining cross-sectional studies provided mean levels of loneliness.

Most of the longitudinal studies also provided mean levels of loneliness, allowing for examining changes in loneliness. Significant increases in loneliness were found during the pandemic compared to pre-pandemic levels. In two studies, mean levels of loneliness significantly increased from pre-pandemic (2018/2019) to during the pandemic (April to July 2020) [60,65]. Houghton et al. [62] found that mean levels varied based on loneliness subscale among Australian adolescents. Compared to pre-pandemic levels, there were significant increases in positive attitudes toward being alone during school closures and school re-openings, but also significant increases in feelings of isolation during school re-openings (but not during school closures). However, Szelei et al. [67] did not find any significant mean-level changes from before school closures to approximately 3 to 6 months later. In their study, the second time point included European adolescents who were still experiencing school closure and others who experienced school re-openings. In some longitudinal studies, loneliness was only assessed at one time point, making it difficult to examine mean-level changes (e.g., [64,68,69]). Other longitudinal studies did not provide formal comparisons across time (e.g., [61,63]).

### 5.1. Associations between Loneliness and Well-Being

#### 5.1.1. Cross-Sectional Results

The most common indicator of well-being examined with loneliness in cross-sectional studies was depression symptoms (14 out of 30). In these studies, a significant positive association was found. Most researchers examined the associations through correlation coefficients which ranged in size such as from *r* = 0.08, *p* = 0.03 [41] to *r* = 0.65, *p* < 0.001 [53]. Other researchers examined the associations through multivariate regression coefficients (e.g., [37,48,56]). In one study, researchers found experiencing extreme isolation (versus not experiencing isolation at all) during the pandemic was associated with the presence of depressive symptoms among Brazilian adolescents (Prevalence Ratio: 2.04, 95% CI [1.00–4.14]; [36]). In these studies, self-report measures were used to assess depression symptoms. In another study, Li et al. [44] found that adolescents in Australia with a previous diagnosis of depression and/or anxiety had a significantly higher mean of loneliness (*M* = 5.63, *SD* = 2.30) compared to adolescents without a previous diagnosis (*M* = 4.56, *SD* = 2.44; *t*(663) = −5.61, *p* < 0.01).

Anxiety symptoms was the next most common indicator of well-being examined with loneliness in cross-sectional studies (12 out of 30). Significant positive associations were found in all these studies, although assessments of anxiety symptoms varied. Most researchers examined loneliness with general anxiety symptoms (e.g., [48,49], whereas fewer researchers examined social anxiety symptoms (e.g., [53]), state anxiety (e.g., [42]), or COVID-19 anxiety (e.g., [31,33]). Most researchers examined the associations through correlation coefficients which ranged in size such as from *r* = 0.20, *p* < 0.001 [44] to *r* = 0.52, *p* < 0.01 [29,53]. Some researchers examined the associations through multivariate regression coefficients (e.g., [29,37,48]).

Several cross-sectional studies (7 out of 30) examined loneliness with overall well-being or mental health. Higher loneliness was significantly associated with poorer overall well-being. In one study, a correlation of loneliness and overall mental health difficulties was *r* = 0.58, *p* < 0.01 for adolescents in China [51]. Wang et al. [57] also found in a sample of Chinese adolescents that loneliness significantly positively predicted overall levels of difficulties (i.e., emotional problems, conduct problems, hyperactivity, peer relationship problems). In another study, the correlation between overall well-being and loneliness-isolation was *r* = −0.52, *p* < 0.001, and between overall well-being and loneliness-proximity was *r* = −0.67, *p* < 0.001 for adolescents in Germany [52]. In a study by Jones et al. [40], adolescents in the United States who felt closer to people at school had lower prevalence rates of poor overall mental health during the pandemic compared to students who did not feel close to people at school (28.4% versus 45.2%). Soneson et al. [55] also looked at prevalence rates and found that 42.2% of children and adolescents in the United Kingdom reported that they felt *slightly* lonelier during the pandemic lockdown and felt that their mental well-being got *worse* and 16% of youth reported that they felt *much* lonelier and that their well-being got *worse*. However, in one study, there were no significant results. Gilsbach et al. [38] examined mean differences in loneliness between children and adolescents with a mental disorder and children and adolescents without a mental disorder in Germany and found no significant differences in loneliness whether using parent-reports, *F*(1, 116) = 1.71, p = 0.19, or self-reports, *F*(1, 137) = 0.25, p = 0.62.

A few researchers examined loneliness with overall internalizing symptoms (e.g., composite of anxiety, depression, and/or somatization symptoms [33,47]; 3 studies out of 31) or externalizing symptoms (e.g., composite of attention problems, aggressive behavior [33]; 2 out of 30). The studies on internalizing symptoms found significant positive correlations, such as ranges from *r* = 0.35, *p* < 0.05 [33] to *r* = 0.90, *p* < 0.001 [47]. The two studies examining externalizing symptoms found mixed results. Dubois-Comtois et al. [33] found that higher aversion to aloneness among children in Canada was positively correlated with externalizing problems, *r* = 0.17, *p* < 0.05, but this association was no longer significant in a multiple regression that included additional predictors such as parent well-being, family functioning, and parent-child relationship, *b* = 0.02, se = 0.08, β = 0.002, p = 0.82, 95% CI [−0.14, 0.18]. Similarly, ADHD diagnosis predicted loneliness when controlling for the child’s age, gender, and other demographic variables, β = 0.22, p < 0.01, but was no longer significant when controlling for other factors such as family cohesion and parental involvement, β = 0.05, p > 0.05 [43].

Six studies (out of 30) examined additional indicators of well-being that were less frequent. Two studies found significant positive associations between loneliness and technology-based pathologies including mobile addiction [45], social media disorder [42], excessive game addiction, and pathological game addiction [59], although these latter two associations varied by children versus adolescents. Palmer et al. [50] and Dondi et al. [32] found significant associations between indicators of loneliness with indicators of sleep related problems, and Palmer et al. [50] additionally looked at post-traumatic stress symptoms. Finally, Jones et al. [40] found that compared to adolescents who did not feel close to people at school, adolescents who felt close to people at school had lower prevalence rates of seriously considering suicide (14.0% versus 25.6%).

#### 5.1.2. Longitudinal Results

Like cross-sectional studies, the most common indicator of well-being examined with loneliness in longitudinal studies was depression symptoms (7 out of 11), followed by anxiety symptoms (4 studies), overall well-being (1 study), internalizing symptoms (2 studies), and externalizing symptoms (2 studies). Several studies found significant within and across time associations between loneliness and depression or anxiety symptoms. Rogers et al. [65] found among adolescents in the United States that loneliness was significantly correlated with depression and anxiety symptoms before the pandemic (October 2019) and during the pandemic (April 2020 during school closure), and all the across time correlations were significant. Similarly, Zuccolo et al. [70] found among Brazilian children and adolescents that baseline levels of loneliness assessed during June to November 2020 predicted higher depression and anxiety symptoms concurrently and at follow-up assessed until June 2021 [70]. Houghton et al. [62] found that four subscales of loneliness (i.e., quality of friendship, feelings of isolation, positive attitudes toward being alone, negative attitudes toward being alone) were mostly all significantly correlated with depression symptoms, internalizing symptoms, externalizing symptoms, and overall well-being within time whether assessed six months before lockdown, during school closures in March 2020, or after school reopening among adolescents in Australia. Across time, generally the findings indicated that feeling more socially connected mitigated the adverse effects of lockdown.

For other studies, the specific pattern varied by type of design and analysis. Alt et al. [60] examined change scores in adolescents in Germany. Higher increases in loneliness from the first time point assessed between 2018 and 2019 to the second time point assessed between May and July 2020 significantly predicted an increase in negative mood (*β* = 0.44, p < 0.001, 95% CI [0.39, 0.54]) and anhedonia (*β* = 0.38, p < 0.001, 95% CI [0.37, 0.54]) across those same two time points. Magson et al. [64] also examined change scores, specifically changes in depression and anxiety symptoms from one year before the pandemic in 2019 to during the pandemic in May 2020 in Australian adolescents. In this study, social disconnection (i.e., indicator of loneliness) assessed during the pandemic significantly moderated the change in depression and anxiety symptoms from before to during the pandemic. Individuals reporting higher social connection during the pandemic reported significantly fewer depression and anxiety symptoms between pre- to during the pandemic. Schwartz-Mette et al. [66] examined additional interaction effects among adolescents in the United States. Self-reported depression and suicide risk pre-pandemic during January to February 2020 (Time 1) interacted with loneliness assessed through EMA during March 2020 (Time 2) to predict depression symptoms in June 2020 during school closures. Specifically, higher loneliness predicted increased depression and suicide risk, in particular for adolescents with higher levels of pre-pandemic depression and suicide risk. In contrast, higher loneliness predicted higher pandemic non-suicidal self-injury (NSSI) frequency for adolescents with lower pre-pandemic NSSI frequency but predicted lower NSSI pandemic frequency among adolescents with higher pre-pandemic NSSI frequency.

There were additional mixed findings and complex nuances within some studies. During March to June of 2020, Cooper et al. [61] found that loneliness was significantly and concurrently positively correlated with internalizing and externalizing symptoms (range *r* = 0.25 to *r* = 0.43, *p* < 0.001). However, in a hierarchical multiple regression, loneliness in March to June of 2020 did not significantly predict these indicators of well-being one month later after controlling for these indicators at the first time point (all *p* > 0.05). Westrupp et al. [68] assessed baseline loneliness using parent-reports in April 2020 to predict trajectories of depression and anxiety symptoms assessed every two to four weeks until May. Overall, the researchers found that loneliness predicted elevated trajectories of these symptoms in areas of Victoria, Australia that were in lockdown and in other areas of Australia that were not in lockdown, but areas of lockdown experienced peaks in child anxiety and depression symptoms.

An additional health outcome examined was prevalence of suicide ideation before the pandemic in 2019 compared to June 2020 when schools reopened in Hong Kong [69]. The researchers found that levels of loneliness during the pandemic was highest among children and adolescents who had newly occurring (i.e., occurrent; 10.7%) or continued (i.e., recurrent; 10.4%) cases of suicide ideation, but these two groups did not significantly differ from one another in loneliness. Finally, one study compared results by clinical status. Houghton et al. [63] examined changes in loneliness pre-pandemic (2019) relative to during school lockdown (March 2020) and post re-opening (July and August 2020) for adolescents with neurodevelopmental disorders (e.g., ADHD, Specific Learning Disorder, Autism Spectrum Disorder) compared to adolescents without neurodevelopmental disorders. Overall, the researchers found few changes in the loneliness subscales of friendship quality and positive and negative attitudes of being alone between adolescents with and without neurodevelopmental disorders. The primary difference between these two groups of adolescents was that adolescents without neurodevelopmental disorders experienced increased feelings of isolation during the lockdown and positive attitudes toward being alone when schools reopened.

### 5.2. Potential Moderating Study Characteristics

We were interested in any study and sample characteristics as potential moderators for the results if provided, such as the timing of data collection (e.g., month/year, before, during, or after school closure), age of participants (e.g., children, adolescents), and underrepresented populations (e.g., clinical populations, minority race/ethnicity and gender/gender identity groups, immigrants and refugees, and geographic regions). One commonly reported potential moderator was timing of data collection. For cross-sectional studies significant associations were generally found with many indicators of well-being. There were 18 out of 31 studies conducted within the first six months of the pandemic (March to September 2020). The other studies were spread out across the remaining months of 2020 (October to December; two studies), 2021 (five studies), 2022 (three studies), and two studies were unclear about when during the pandemic they were conducted. For longitudinal studies, the length of follow-up between time points ranged from one month, three to six months, and one year. There were seven out of 11 studies with a pre-pandemic assessment that generally showed increases in loneliness and/or loneliness as a predictor of poorer well-being (e.g., [62,64,66]). However, in one study an increase in post-traumatic stress symptoms in European adolescents was associated with decreased school belonging, but only for adolescents assessed after school closures [67]. In three studies, the first time point assessed was early in 2020 during the start of the pandemic and in these studies baseline loneliness predicted higher internalizing symptoms (e.g., [61,68,70]). We found that results on other study and sample characteristics such as clinical status, minority race/ethnicity, gender/gender identity groups, immigrants and refugees, and geographic regions were not systematically reported, which prevented us from analyzing results by these factors.

## 6. Discussion

Across the 41 studies from around the world in our systematic review, results on the prevalence of child and adolescent loneliness varied as a function of these studies’ characteristics. Some of the cross-sectional studies found over half of children and adolescents felt lonely at least some of the time if not more (e.g., 41.4% moderately, 20.1% very, 8.5% extremely [36]). Many researchers also found increases in mean levels of loneliness from before to during the pandemic [60,62,65], but in one study there were no significant mean-level changes from school closures to three to six months later [67]. It is important to note that in this latter study, the first time point was early in the pandemic and not pre-pandemic. Thus, much of the contradictory findings on the associations and prevalence of loneliness could be due to the timing of assessments and study design. These mixed findings indicate that timing and number of assessments must be considered when examining pandemic related loneliness and well-being among children and adolescents. Buecker and Horstman [17] note that heterogeneity in follow-up assessment points can complicate comparisons of studies. Overall, these loneliness rates are largely consistent with pre-pandemic patterns of increases in adolescent loneliness [9].

We also found significant associations between loneliness and indicators of well-being among children and adolescents during the COVID-19 pandemic. Recent systematic reviews on pre-pandemic studies indicate significant associations between loneliness and indicators of well-being such as anxiety and depression symptoms, in both clinical [27] and non-clinical [16] samples. Systematic reviews of studies from early in the pandemic also found similar results of pandemic impacts on child and adolescent depression and anxiety symptoms [22,23,24]. Our systematic review extends these findings further by integrating the literature across these two fields. All cross-sectional studies examining loneliness with depression symptoms, anxiety symptoms, and additional forms of internalizing symptoms found significant positive associations that ranged from small to moderate in effect size. The two cross-sectional studies on externalizing symptoms also found consistent, although non-significant results. At the univariate level, there were significant correlations between loneliness and externalizing symptoms, but when additional factors were controlled for such as demographic variables, parent, and family factors, the association was no longer significant in both studies [33,43].

In contrast, results from longitudinal studies were more complex and depended on timing, the number of loneliness assessments, and other factors controlled for in statistical analyses. Most of the studies found that loneliness was significantly associated with anxiety and depression symptoms before and during the COVID-19 pandemic (e.g., [65,70]), and that social connection mitigated the negative impact of pandemic-related lockdown on mental well-being [62]. However, several studies revealed some complex interactive effects such that more adverse impacts of loneliness were evident for adolescents with poorer pre-pandemic well-being [64,66]. In other studies, when controlling for indicators of well-being from the first three months of the pandemic, loneliness did not significantly predict these aspects of well-being one month later [61].

We also found that there was limited diversity in study designs and sample characteristics, preventing a thorough examination of potential moderators. Most studies were from Europe followed by the United States and Australia. In addition, only four studies explicitly examined clinical samples and these results varied. Some researchers found that loneliness did not vary by clinical status (e.g., [38]) and other researchers found mixed results depending on the aspects of loneliness examined [63]. A more diverse and systematic approach to examining multiple underrepresented samples (e.g., clinical populations, minority race/ethnicity and gender/gender identity groups, LGBTQ+, immigrants and refugees, Indigenous peoples, and rural and northern geographic regions) should be included in future studies to better understand these patterns. Indeed, researchers have noted that underrepresented youth populations were disproportionately impacted by the pandemic [24]. For example, many children and adolescents requiring special services for disabilities and or school-based healthcare lost access due to in-person school closures.

Most studies were also on adolescents, with 11 studies combining child and adolescent samples and only three studies examining child samples. In a recent meta-analysis of pre-pandemic studies on loneliness and depression symptoms, age was not a significant moderator [71]. The researchers recognized that one explanation may be due to low power to detect these effects due to a smaller number of studies on child samples. Nearchou et al. [22] noted potential age-related differences in the pandemic’s impact. Older children may be impacted directly by school closures, whereas younger children may be impacted by pandemic-related stress placed on caregivers. Finally, although many studies mentioned the timing of study assessments, few studies explicitly accounted for school closures [62,67]. Accordingly, we primarily drew conclusions on assessment timing rather than school closures. Given that many published studies were conducted within the first six months of the pandemic, additional longitudinal studies that include multiple assessments of loneliness and well-being and underrepresented youth populations are needed. Through these studies, we can better understand the accumulation of pandemic-related stressors and the long-term impact of the pandemic on loneliness and well-being, and specifically when, for who, and for how long.

Results from our systematic review have several key implications for practice and policy. Ways to build meaningful social connections that will help improve the well-being of children and adolescents should be a priority. This does not only include practices and policies in education (i.e., schools), but in sectors beyond education including in health, community, and social services. It is clear in studies with pre-pandemic baseline assessments that there was an already existing problem surrounding the well-being of youth before the pandemic. Moreover, in a recent meta-analysis of loneliness interventions of individuals between ages 3 and 25, it was evident that few targeted interventions exist for child and adolescent loneliness [72]. Across 39 studies, the researchers found that some of the intervention designs that showed the largest reductions in loneliness focused on social and emotional learning skills. Social and emotional learning programs have shown promising results before the pandemic [73] and have been encouraged during the pandemic [74]. These programs can help reduce loneliness as well as improve academic goals since learning occurs well within supportive relationships [75].

Several researchers have also proposed prevention and intervention efforts that focus on opportunities to increase the number of social relationships, and more importantly, the connection and quality of these relationships [23]. Methods of increasing children’s and adolescents’ feelings of belonging to a social group and experiencing positive social rewards can include access to physical exercise, social hobbies, and entertainment [25,71]. This is in line with additional findings by Eccles and Qualter [72] where randomized control trials of interventions focused on learning a new hobby also helped to reduce loneliness. During the pandemic, many extracurricular activities and physical activities were limited or cancelled [76]. Recently, in a study comparing a cohort of adolescent athletes during May 2020 (i.e., during pandemic related sport closure) to another cohort of adolescent athletes during May 2021 (i.e., during sport re-openings), the prevalence of depression symptoms decreased between these two time points [77]. Accordingly, efforts to rejoin these social networks, extracurricular activities, and physical activities may help reduce loneliness and adverse well-being, but caution should be applied to ensure that practices are equitable, culturally sensitive, and tailored to the children and adolescents involved [78].

Despite the results and implications of our systematic review, there are a few limitations to our review method. First, our search terms included loneliness and indicators of social isolation, belonging, and mattering to stay consistent with some previous systematic reviews, despite these constructs being distinct (e.g., [16,27]). The final articles selected may have differed if we restricted studies to include only loneliness. Second, our search terms for indicators of well-being were largely based on previous studies and reviews, such as well-being, mental health, depression, anxiety, internalizing symptoms, externalizing symptoms, and substance abuse. However, we found that additional indicators of well-being were assessed in studies, such as post-traumatic stress symptoms, sleep disorder symptoms, and online game addiction. Although these specific topics were beyond our scope, inclusion of additional search terms could reveal a greater number of patterns in future systematic reviews. For example, the impact of social media use on loneliness and well-being can vary because of pandemic restrictions that prevent in-person interaction and in turn facilitate online social interactions (e.g., [79]). Third, we kept our initial search age range of children and adolescents between 0 and 18 to be consistent with previous systematic reviews and meta-analyses (e.g., [21]). However, this is a large age range and future reviews can focus on either children or adolescents combined with searches on specific sample characteristics such as clinical populations, race/ethnicity, and gender/gender identity to identify more detailed results. Finally, results from the included studies cannot infer cause and effect relationships among the pandemic, loneliness, and well-being. Some of the longitudinal studies demonstrated changes in loneliness in relation to changes in well-being, but they did not test causal relationships. There are many other factors that need to be examined, such as family relationships, parent or caregiver well-being, and socioeconomic and financial stressors from before and during the pandemic (e.g., [33]).

Across the cross-sectional and longitudinal studies in our systematic review of loneliness and well-being in children and adolescents across the world, loneliness was quite prevalent during the pandemic. Over half of children and adolescents in several studies reported at least some feelings of loneliness at some point during the pandemic. In other studies, mean levels of loneliness increased from pre-pandemic levels. Loneliness was also related to poorer well-being including higher depression symptoms, anxiety symptoms, and mental health difficulties. Supportive, healthy, and meaningful social relationships should be prioritized across sectors. The characteristics of existing effective strategies with respect to social and emotional learning programs and school-based mental health can be integrated with newer equitable practices that are culturally sensitive to underrepresented child and adolescent populations. Through integrating evidence with practice and policy, we can best help children and adolescents in ways that are sustainable both during and beyond the COVID-19 pandemic.

## Figures and Tables

**Table 1:** Summary of Cross-Sectional Studies.

	Sample			Measures		Results		Additional Characteristics	
Author, Country	Type	*N* (% Males/Boys)	Age Range; *M (SD)*; Type	Loneliness Measure	Well-Being Measure(s)	Loneliness *M (SD*) or *N* (%)	Association between Loneliness and Well-Being	Data Collection Timing/School Closure	Qual (/9)
Boursier et al. [31], Italy	Com	544 (28.1)	13–20; 16.22 (1.83); Adol	Italian Loneliness Scale-self-report	Depression subscale of Depression-Anxiety Stress Scale-21 Multidimensional Assessment of COVID-19 related fears -self-report	2.11 (0.6) Min = 1 Max = 4	Dep: *r* = 0.564, *p* < 0.001 Loneliness→Depression β = 0.721, *se* = 0.054, *p* < 0.001 COVID-19 Anx: *r* = 0.294, *p* < 0.001 COVID-19 Anx→Loneliness β = 0.198, *se* = 0.302, *p* < 0.001	January–March 2021, school closure unclear	6
Cauberghe et al. [29], Belgium	Com	2165 (34.4)	13–19; 15.51 (1.59); Adol	Revised UCLA Loneliness Scale-6 items-self-report	General Anxiety Disorder Scale-7 Center of Epidemiological Studies Depression Scale -self-report	Boys: 2.62 (0.76) Girls: 2.92 (0.77)	Anx: *r* = 0.523, *p* < 0.01 Happiness (vs. Depression): *r* = −0.590, *p* < 0.01 Loneliness→Happiness β = −0.616, *p* < 0.01	16–30 April 2020, during school closure	6
Dondi et al. [32], Italy	Both	6210 (NA)	Up to age 18; NA; Both	Single item of child feeling lonely-parent-report	Single items from Sleep Disturbance Scale for Children -difficulty falling asleep (sleep disorder) -maintaining sleep -nightmares -parent-report	No: 32.4% Yes, not putting it into words: 31.6% Yes, putting it into words: 36.0%	Loneliness→Sleep Falling asleep: -Yes not putting into words: *OR:* 1.85 (*se* = 0.14) -Yes putting into words: *OR:* 1.97 (*se* = 0.15) Staying Asleep: -Yes not putting into words: *OR:* 1.83 (*se* = 0.16) -Yes putting into words: *OR:* 2.11 (*se* = 0.18) Nightmares: -Yes not putting into words: *OR:* 2.10 (*se* = 0.21) -Yes putting into words: *OR:* 2.05 (*se* = 0.20) *p* < 0.01 for all	1 September–15 October 2020, school closure unclear	5
Dubois-Comtois et al. [33], Canada	Com	144 (51.4)	9–12; 10.44 (1.09); Both	Negative Experienced Aloneness Scale of the Loneliness and Aloneness Scale for Children and Adolescents-self-report	Fear of COVID-19 Scale (anxiety toward COVID-19)-self-report Child Behavior Checklist (externalizing)-parent-report Youth-Self Report (internalizing)-self-report	33.65 (6.88) Min = 12 Max = 48	Aversion to Aloneness and: Internalizing: *r* = 0.35, *p* < 0.001 Loneliness→Internalizing *b* = 0.27, *se* = 0.08, β = 0.26, *p* = 0.001, 95% *CI* [0.11, 0.43] Externalizing: *r* = 0.17, *p* < 0.05 Loneliness→Externalizing *b* = 0.02, *se* = 0.08, β = 0.002, *p* = 0.82, 95% *CI* [−0.14, 0.18] COVID-19 Anx: *r* = 0.32, *p* < 0.001 Loneliness→COVID-19 Anx *b* = 0.22, *se* = 0.07, β = 0.26, *p* = 0.002, 95% *CI* [0.08, 0.35]	18 April–18 May 2020, during school closure	5
Ellis et al. [34], Canada	Com	1054 (21.9)	14–18; 16.68 (0.78); Adol	Revised UCLA Loneliness Scale-self-report	Brief Symptom Inventory-self-report	Boys: 2.26 (*se* = 0.15) Girls: 2.85 (*se* = 0.06) Min = 1 Max = 4	Dep: *r* = 0.53, *p* < 0.01	4–16 April 2020, during school closure	6
Espinoza and Hernandez [35], US	Com	993 (41)	14–18; 16.09 (1.24); Adol	Items adapted from Asher and Wheeler Loneliness Scale-self-report	Center for Epidemiologic Studies Depression Scale-self-report	2.56 (1.03) Min = 1 Max =5	Dep: *r* = 0.61, *p* < 0.001	15 April–May 2020; school closure unclear	6
Fernandes et al. [36], Brazil	Com	343 (44.3)	14–18; 16.1 (0.9); Adol	COVID-19 related measure with social isolation item-self-report	Children’s Depression Inventory-self-report	Social Isolation: -Extremely isolated: 8.5% -Very isolated: 20.1% -Moderately isolated: 41.4% -Slightly isolated: 19.8% -Not at all: 10.2%	Isolation (vs. No Isolation)→Dep (vs. No Dep) *PR* (Prevalence Ratio): 2.04, 95% CI [1.00–4.14]	April–July 2021, school closure unclear	6
Fogarty et al. [37], Australia	Com	257 (45.5)	14–17; 15.7 (0.87); Adol	COVID-19 CRISIS Tool, single item on feeling lonely -self-report Scale of 0–4 dichotomized: 0–1 = 0, 2–4 = 1	Patient Health Questionnaire-Adolescent The Generalised Anxiety Disorder Scale-7 -self-report	38.7% said moderately to extremely lonely	Dep: *r* = 0.56, *p* < 0.001 Loneliness→Dep β = 0.31, *b* = 3.88, 95% *CI* [2.61, 5.15], *p* < 0.001 Anxiety: *r* = 0.44, *p* < 0.001 Loneliness→Anx β = 0.16, *b* = 1.44, 95% *CI* [0.44, 2.43], *p* = 0.005	July–September 2022 (during second lockdown), during school closure	7
Gilsbach et al. [38], Germany	Both	195 147 Clinical Group with Diagnosed Mental Disorder (43.6) 48 Non-Clinical (66.7)	6–18; Clinical: 13.3 (3.0) Non-Clinical: 13.5 (3.0); Both	Single item on impact of COVID-19 on loneliness -parent-report -self-report	NA; compared clinical to non-clinical sample	*M (SD)* NA Min = −2 (not at all) Max = 2 (very much)	Mean difference in Loneliness between Clinical and Non-Clinical samples: -Self-report: *F*(1, 137) = 0.25, *p* = 0.62 -Parent-report: *F*(1, 116) = 1.71, *p* = 0.19	Spring 2020, school closure unclear	6
Hou et al. [39], China	Com	826 (57.1)	Senior high school up to 18; NA; Adol	UCLA Loneliness Scale-self-report	Patient Health Questionnaire-9-self-report	47.92 (8.55) Min = 0 Max = 60	Dep: *r* = 0.425, *p* < 0.001 Loneliness→Dep β = 0.337, *p* < 0.001,	April–May 2020, school closure unclear	7
Jones et al. [40], US	Com	7705 (49.6)	Grades 9 to 12; NA; Adol	Single item about feeling close to persons at school-self-report	Single items: -how often mental health not good during pandemic -how often mental health not good during last 30 days -considering suicide past 12 months -attempt suicide past 12 months -self-report	Agree, felt close: 46.6%Not sure or disagree: 53.4%	Overall: Compared to students who did not feel close to persons at school, students who felt close: -Lower prevalence of poor mental health during pandemic: 28.4% vs. 45.2% -Lower prevalence of poor mental health last 30 days: 23.5% vs. 37.8% -Lower prevalence of having seriously considered suicide: 14.0% vs. 25.6% -Lower prevalence of having attempted suicide: 5.8% vs. 11.9% *p* < 0.05 for all	January–June 2021, school closure unclear	5
Kayaoğlu and Başcıllar [41], Turkey	Com	423 (36.4)	10–19; 15.23 (2.23); Adol	UCLA Loneliness Scale-short form-self-report	Children’s Depression Inventory-self-report	16.43 (4.93) Min = 7 Max = 52	Dep: *r* = 0.084, *p* = 0.03	Not provided, but during the COVID-19 Pandemic	8
Kilinçel and Muratdagi [42], Turkey	Com	1142 (63.2)	12–18; 15.6 (2.8); Adol	UCLA Loneliness Scale-self-report	State-Trait Anxiety Inventory-self-report Social Media Disorder Scale-self-report	NA	State Anx: *r* = 0.380, *p* < 0.01 Social Media Disorder: *r* = 0.093, *p* < 0.01 Loneliness→Social Media Disorder β = 0.150, *p* = 0.001	29–30 March 2022, online school assessed	5
Laslo-Roth et al. [43], Israel	Both	280; ADH-D: 166 (71) No ADH-D: 114 (57)	NA; ADHD: 9.33 (2.45) No ADHD: 9.89 (2.2); Child	Single item of child seeming to be lonely-parent-report	ADHD diagnosis	2.71 (1.27) Min = 1 Max = 5	ADHD status→Loneliness Step 1: β = 0.22, *p* < 0.01 Step 2: β = 0.05, NS	During COVID-19 pandemic but details not provided, school closure unclear	6
Li et al. [44], Australia	Com	760 (28)	12–18; 14.8 (1.26); Adol	Single item from UCLA Loneliness Scale-self-report	Asked if previously diagnosed with anxiety and/or depression Warwick Edinburgh Mental Well-Being Scale short-form Body Preoccupation Scale of the Illness Attitude Scales -self-report	Frequency of feeling alone: -Hardly ever: 17.1% -Some of the time: 30.7% -Often: 51.4%	Mean Loneliness by Diagnosis vs. No Diagnosis: *t*(663) = −5.61, *p* < 0.01 -Diagnosis: *M* = 5.63, *SD* = 2.30 -No Diagnosis: *M* = 4.56, *SD* = 2.44 Loneliness and: Health Anx: *r* = 0.20, *p* < 0.01 Overall Well-Being: *r* = −0.59, *p* < 0.01	2 June–5 August 2020, during school closure	4
Li et al. [45], China	Com	1034 (61.2)	12–19; 15.76 (1.20); Adol	UCLA Loneliness Scale-self-report	Mobile Phone Addiction Index-self-report	44.88 (10.19) Min = 20 Max = 100	Mobile Addiction: *r* = 0.14, *p* < 0.001 β = 0.14 *se* = 0.03, *p* < 0.001,	22–29 May 2020, school closure unclear	7
Liu et al. [46], China	Com	1594 (49.4)	9–16; 13.13 (1.54); Both	Loneliness and Aloneness Scale for Children and Adolescents -Peer loneliness -Family Loneliness-self-report	Center for Epidemiologic Studies Depression Scale for Children-self-report	-Peer Loneliness: 1.32 (0.46) -Family Loneliness: 1.78 (0.68) Min = 1 Max = 4	Peer loneliness and dep: *r* = 0.49, *p* < 0.001 Family loneliness and dep: *r* = 0.58, *p* < 0.001	April 2020, school closure unclear	6
Low and Mounts [47], US	Com	272 (NA)	12–18; NA; Adol	UCLA Loneliness Questionnaire—parent-report	Revised Child Anxiety and Depression Scale-parent-report	0.95 (0.73) Min = 0 Max = 3	Internalizing: *r* = 0.90, *p* < 0.01	June–November 2020, during school closure	6
Murata et al. [48], US	Com	583 (20)	NA; 15.80 (1.40); Adol	Feelings of loneliness since the COVID-19 pandemic-self-report	Patient Health Questionnaire Generalized Anxiety Disorder (GAD-7) -self-report	6.9 (2.3) Min = 1 Max = 10	Loneliness→Dep: β = 0.376, 98% *CI* [0.24, 0.52], *p* < 0.001, Loneliness→Anx: β = 0.57, 98%CI [0.41, 0.74], *p* < 0.001,	27 April–13 July 2020; school closure unclear	5
Oosterhoff et al. [49], US	Com	683 (22.7)	13–18; 16.35 (1.13); Adol	Interpersonal Needs Questionnaire (Belongingness)-self-report	Patient-Reported Outcomes Measurement Information-short-fixed-form -Depression scale -Anxiety scale -self-report	4.21 (1.49) Min = 1 Max = 7	Dep: *r* = −0.45, *p* < *0.05* (depression related to higher belongingness) Anx: *r* = −0.21, *p* < *0.05* (anxiety related to higher belongingness)	29–30 March 2022; school closure unclear	7
Palmer et al. [50], US	Com	459 (18.7)	13–18; 16.24 (1.26); Adol	Single item on how lonely from Positive and Negative Affect Schedule-X-self-report	Positive and Negative Affect Schedule-for children UCLA Post-Traumatic Stress-Reaction Index Pittsburgh Sleep Quality Index National Sleep Foundation Sleep Diary Consensus Sleep Diary Single item on bad dreams -self-report	2.82 (1.36) Min = 1 Max = 5	Internalizing: Positive Affect: *r* = −0.33, *p* < 0.01 Negative Affect: *r* = 0.63, *p* < 0.01 Others: Post-Traumatic Stress-Symptoms: *r* = 0.61, *p* < 0.01 Nightmares: *r* = 0.12, *p* < 0.01 Sleep onset: *r* = 0.17, *p* < 0.01 Sleep quality: *r* = 0.21, *p* < 0.01	1–5 April 2020, during school closure	7
Pan et al. [51], China	Com	5783 (60.75)	NA; NA; Adol	UCLA Loneliness Questionnaire-self-report	Mental Health of Middle School Students-self-report (higher scores mean worse mental health)	42.77 (8.75) Min = 20 Max = 80	Overall Mental Health: *r* = 0.578, *p* < 0.01 Loneliness→Overall Mental Health β = 0.036, *p* < 0.001	May 2020, during school closure	4
Pfetsch et al. [52], Germany	Com	205 (43)	14–19; 15.83 (1.44); Adol	UCLA-Revised (10 items) subscales: -feelings of isolation -lack of proximity -self-report	Short Warwick-Edinburgh Mental Well-Being Scale-self-report	NA	Overall Well-Being: -Loneliness isolation: *r* = −0.67, *p* < 0.001 Isolation→Well-being Model 3: *b* = −0.21, *se* = 0.07, β = −0.28, *p* < 0.001 Model 4: *b* = −0.20, *se* = 0.07, β = −0.26, *p* < 0.01 -Loneliness proximity: *r* = −0.52, *p* < 0.001 Proximity→Well-being Model 3: *b* = −0.10, *se* = 0.07, β = −0.10, *p* > 0.05 Model 4: *b* = −0.09, *se* = 0.07, β = −0.10, *p* > 0.05	Middle of March–beginning April 2021, during school closure	7
Sette et al. [53], Italy	Com	748 (48.3)	7–11; 8.91 (1.07); Child	Loneliness and Social Dissatisfaction Questionnaire-self-report	Children’s Depression Inventory Social Anxiety Scale for Children-Revised -self-report	1.47 (0.67) Min = 1 Max = 5	Dep: *r* = 0.65, *p* < 0.001 Social Anx: *r* = 0.52, *p* < 0.001	December 2020–April 2021, both school closure and reopened assessed	7
Sette et al. [54], Italy, Spain, UK	Com	236 (44.49)	6–12; 9.25 (1.2); Child	Adapted from Asher et al.-self-report	The Children’s Depression Inventory-Short Form Spence Children’s Anxiety Scale (SCAS-Child)-self-report	1.57 (0.57) Min = 1 Max = 5	Dep: *r* = 0.62, *p* < 0.001 Anx: *r* = 0.41, *p* < 0.001	23 April–25 June 2020, during school closure	6
Soneson et al. [55], UK	Com	16,940 (38.9)	8 to 18; NA; Both	-General loneliness (reference) -Change in loneliness during lockdown -self-report	Single item on mental well-being	NA	General Loneliness (Never, Sometimes, Often) by Mental Well-Being (Worse, Same, Better) -Never and worse: 22.8% -Never and same: 61.0% -Never and better: 61.3% -Sometime and worse: 47.5% -Sometimes and same: 31.9% -Sometimes and better: 32.2% -Often and worse: 30.0% -Often and same: 7.2% -Often and better: 6.5% Change in Loneliness (Much Less, Slightly Less, Same, Slightly More, Much More) by Mental Well-Being (Worse, Same, Better) -Much less and worse: 5.7% -Much less and same: 9.3% -Much less and better: 23.7% -Slightly less and worse: 11.7% -Slightly less and same: 14.1% -Slightly less and better: 21.0% -Same and worse: 24.4% -Same and same: 55.2% -Same and better: 34.7% -Slightly more and worse: 42.2% -Slightly more and same: 18.7% -Slightly more and better: 18.1% -Much more and worse: 16.0% -Much more and same: 2.7% -Much more and better: 2.5%	May–July 2020, both school closure and reopened assessed	4
Varela et al. [56], Chile	Com	2370 (18.27) total but only subsample 15–18	15–18; NA; Adol	Single item on fear of loneliness-self-report	Patient Health Questionnaire-9-self-report	N/A Min = 0 Max = 3	Fear of Loneliness→Dep Dep: *b* = 3.790 [4.358], *se* = (0.196, [0.161]), *p* < 0.001 (brackets = weighted values)	December 2020, school closure unclear	8
Wang et al. [57], China	Com	6587 (49.9)	NA; 15.50 (1.70); Adol	Single item on loneliness from Children’s Depression Inventory-self-report	Strengths and Difficulties Questionnaire-self-report	No: 66.1% Yes = 33.9%Min = Never Max = Many times or always	Overall Well-Being: (higher difficulties) Loneliness→Difficulties Model 1: *b* = 5.41, *se* = 0.25, β = 0.45, *p* < 0.001 Model 2: *b* = 5.95, *se* = 0.20, β = 0.50, *p* < 0.001 Model 3: *b* = 6.23, *se* = 0.18, β = 0.52, *p* < 0.001	16 April–14 May 2020, during school closure	5
Yilmaz et al. [58], Turkey	Com	3655 (39.4)	Grade 6 to 11; NA; Adol	Developed a scale that included Loneliness Subscale-self-report (4 items)	Developed a scale that included Anxiety Subscale-self-report (4 items)	2.96 (1.19) Min = 1 Max = 5	Anx: *r* = 0.353, *p* < 0.001	June 2020, during school closure	4
Zhu et al. [59], Hong Kong	Com	1346 (47.3)	8–17; 12.6 (1.32); Both	Single item about feeling lonely-self-report	Game Addiction Scale-self-report	0.52 (0.87) Min = 0 Max = 3	Loneliness→Game Addiction Primary Students: -Excessive Game Addiction: a*OR:* 0.71, 95% CI [0.65–0.78], *p* < 0.001 -Pathological Game Addiction: a*OR:* 0.94, 95%CI [0.80, 1.12], *p* > 0.05 Secondary Students: -Excessive Game Addiction: a*OR:* 1.00, 95%CI [0.95–1.05], *p* > 0.05 -Pathological Game Addiction: a*OR:* 0.88, 95% CI [0.9–0.99], *p* < 0.01.	June 2020, schools reopened	6

Note. When multiple statistics were reported for results, either the main univariate/multivariate results are reported or a summary description of the main pattern of results are reported; NA = Not Applicable or Not Provided; Both (under sample type) = Both community and clinical sample; Com = Community sample; Adol = Adolescent sample; Child = Child sample; Both (under sample age) = Both child and adolescent sample; Min = Minimum score; Max = Maximum score; Dep = Depression Symptoms; Anx = Anxiety Symptoms; Internalizing = Internalizing Symptoms; Externalizing = Externalizing Symptoms; ADHD = Attention Deficit Hyperactivity Disorder; Qual = Quality Rating.

**Table 2:** Summary of Longitudinal Studies.

	Sample			Measures		Results		Study Characteristics	
Author, Country	Type	*N* (% Males/ Boys)	Age Range; *M (SD)*; Type	Loneliness Measure	Well-Being Measure(s)	Loneliness *M (SD)* or *N* (%)	Association between Loneliness and Well-Being	Data Collection Timing/School Closure	Qual (/10)
Alt et al. [60], Germany	Com	843 (42.7)	14–17; 16.11 (0.78) Adol	Single item from UCLA Loneliness Scale-self-report	State-Trait Depression Scale -Anhedonia -Negative Mood -self-report	T1: 2.10 (1.15) T2: 2.27 (1.30) Min = 1 Max = 5 Change significant at *p* < 0.001	T1: Loneliness and: -Anhedonia: *r* = 0.51, *p* < 0.01 -Negative Mood: *r* = 0.55, *p* < 0.01 T2: Loneliness and: -Anhedonia: *r* = 0.44, *p* < 0.01 -Negative Mood: *r* = 0.53, *p* < 0.01 Change in T1 to T2 Loneliness with: Change in T1 to T2 Anhedonia: *r* = 0.32, *p* < 0.01 Change in T1 to T2 Negative Mood: *r* = 0.40, *p* < 0.01 Summary: Higher rise in loneliness→stronger increase in: -Negative Mood: β = 0.44, *p* < 0.001, *r* = 0.49, 95% *CI* [0.39, 0.54] -Anhedonia: β = 0.38, *p* < 0.001, *r* = 0.43, 95% *CI* [0.37, 0.54]	T1: October 2018–August 2019 T2: May 2020–July 2020, during both school closure and reopening	9
Cooper et al. [61], UK	Com	Total: 894 T1: 451 (51.2) T2: 443 (49.9)	11–16; T1: 13.37 (1.64) T2: 13.28 (1.68); Adol	UCLA Short Loneliness Scale-self-report	Strengths and Difficulties Questionnaire -Internalizing -Externalizing -self-report	Total: T1: 6.85 (20.01) Longitudinal Group: T1: 6.65 (1.92) T2: 6.75 (2.06) Min = 4 Max = 12	T1: -Internalizing: *r* = 0.43, *p* < 0.001 -Conduct Problems: *r* = 0.25, *p* < 0.001 -Hyperactivity-Inattention: *r* = 0.27, *p* < 0.001 T1 Loneliness→T2 (controlling for T1 well-being): -Internalizing: β = 0.02, 95% *CI* [−0.05, 0.09], *p* = 0.568 -Conduct Problems: β = 0.03,95% *CI* [−0.04, 0.11], *p* = 0.384 -Hyperactivity-Inattention: β = 0.00, 95% *CI* [−0.07, 0.07], *p* = 0.983	T1: March to June 2020 T2: 1 month later (some during lockdown, some after), both closure and reopen assessed	6
Houghton et al. [62], Australia	Com	785 (41.2)	10–17; 14.1(1.31); both	Perth A-Loneness Scale: -Quality of friendship -Feelings of isolation -Positive attitudes toward being alone -Negative attitudes toward being alone -self-report	Children’s Depression Inventory-2 short version Warwick-Edinburgh Mental Well-Being Scale Strengths and Difficulties Questionnaire -Internalizing -Externalizing -self-report	T2: -Isolation: 10.38 (4.73) -Friendship: 27.78 (6.22) -Positive: 20.75 (5.98) -Negative:17.93 (5.56) T3: -Isolation: 10.67 (5.03) -Friendship: 27.85 (6.33) -Positive: 21.29 (6.04) -Negative:18.05 (5.67) T4: -Isolation: 10.67 (5.03) -Friendship: 27.65 (6.41) -Positive: 220.01 (5.75) -Negative:17.66 (5.42) Min = 6 Max = 36 Summary: Compared to T2 (Pre-COVID-19): -increase in positive attitudes at T3 (closure) and T4 (reopening) -increase in isolation at T4 but not T3 -no change in friendship and negative attitudes	Summary of within time correlations: -Dep and Internalizing: isolation (+), friendship (-), positive attitudes (+), negative attitudes (-) -Overall Well-Being: isolation (-), friendship (+), positive attitudes (-) except at T3 and T4; negative attitudes (-) -Externalizing: isolation (+), friendship (-), negative attitudes (+) Summary of across time associations: -T2→T3 (pre-COVID-19 to lockdown) and T3→T4 (lockdown to reopening) found friendship and positive attitudes associated with better well-being (e.g., depression, overall well-being)	T1: 18 months pre-COVID-19 lockdown T2: 6 months pre-COVID-19 lockdown T3: March 2020, school closure T4: 3 months after school reopening; Both closure and reopening assessed	9
Houghton et al. [63], Australia	Both	476 NDD: 238 (55.0)No NDD: 238 (55.0)	10–16; NA; Both	Perth A-Loneness Scale: -Quality of friendship -Feelings of isolation -Positive attitudes toward being alone -Negative attitudes toward being alone -self-report	N/A (NDD vs. No NDD)	*M (SD)* NA Min = 6 Max = 36	Summary of change in loneliness by NDD and no NDD with T2 as reference: Isolation: -T3: NDD no change, no NDD increase -T4: NDD/no NDD no change Friendship: -T3 and T4: NDD/no NDD no change Positive attitudes: -T3: NDD/no NDD no change -T4: NDD no change, no NDD increase Negative attitudes: -T3 and T4: NDD/no NDD no change	T1: Pre-COVID-19 November 2018 T2: Pre-COVID-19 April–May 2019 T3: March 2020, school closure T4: 4 weeks after school reopening July–August 2020, both school closure and reopen assessed	8
Magson et al. [64], Australia	Com	248 (49.2)	13–16; 14.4 (0.5); Adol	T2: The Social Connectedness Scale-self-report	T1 and T2: Depression: Short Mood and Feelings Questionnaire-Child Version General Anxiety subscale of Spence Children’s Anxiety Scale -self-report	2.92 (1.29) Min = 1 Max = 6	T2 Social Disconnection and Dep: T1: *r* = 0.35, *p* < 0.001 T2: *r* = 0.62, *p* < 0.001 T2 Social Disconnection and Anx: T1: *r* = 0.33, *p* < 0.001 T2: *r* = 0.47, *p* < 0.001 Social Disconnection as moderator predicting difference between: T1 Dep→T2 Dep: *F* = 42.06, *R*^2^ = 0.146, β = −0.95, *t*(245) = 5.41, *p* < 0.001 T1 Anx→T2 Anx: *F* = 10.27, *R*^2^= 0.040, β = −0.54, *t*(246) = −3.20, *p* < 0.001 Summary of moderation: social disconnection moderated change in depression and anxiety from T1 to T2; higher social connection during the COVID-19 pandemic reported fewer depressive symptoms and anxiety from T1 to T2	T1: 2019, 12 months before COVID-19 T2: 5–14 May 2020 (2 months after stay-at-home order), during school closure and online school assessed	9
Rogers et al. [65], US	Com	407 (50.1)	14–17; 15.42 (1.16); Adol	Three-item Loneliness Scale-self-report	Children’s Depression Inventory-short version Generalized Anxiety Disorder Scale -self-report	T1: 1.30 (0.47) T2: 1.44 (0.53) Min = 1.00 Max = 3.00 Change significant: *t*(406) = 5.52, *p* < 0.001;	Dep: -Within time: T1 *r* = 0.69; T2 *r* = 0.64 -Across time: T1 Dep with T2 Loneliness *r* = 0.50; T2 Dep with T1 Loneliness *r* = 0.54 Anx: -Within time: T1 *r* = 0.71; T2 *r* = 0.62 -Across time: T1 Anx with T2 Loneliness *r* = 0.50; T2 Anx with T1 Loneliness *r* = 0.42 all *p* < 0.001	T1: October 2019 T2: 11–25 April 2020, during school closure	8
Schwartz-Mette et al. [66], US	Com	362 (33)	Middle school students: 12.61 (0.93)High school students: 16.04 (1.16); Adol	T2: Ecological Momentary Assessment (EMA) of loneliness 3 times a day for 7 days-self-report	T1 and T3: Center for Epidemiological Studies-Depression scale Non-Suicidal Self-Injury (NSSI) adapted from Prinstein et al. T1 and T3 Suicide Behaviors Questionnaire-Revised T2: EMA of COVID-19 Health Anxiety	2.82 (1.16) Min = 1 Max = 5	Dep: T1: *r* = 0.48, *p* < 0.001 T3: *r* = 0.60, *p* < 0.001 NSSI: T1: *r* = 0.20, *p* < 0.001 T3: *r* = 0.19, *p* < 0.01 Suicide Risk: T1: *r* = 0.38, *p* < 0.001 T3: *r* = 0.38, *p* < 0.001 Health Anxiety: *r* = 0.34, *p* < 0.001 Summary of Interactions: T2 loneliness moderated: -T1 depression→T3 depression and T1 suicide risk→T3 suicide risk for individuals with higher levels at T1 -T1 NSSI→T3 NSSI for individuals with lower NSSI at T1	T1: January/February 2020 T2: March 2020 (EMA), during COVID-19 T3: June 2020, during school closure	5
Szelei et al. [67], European Countries	Com	Total: 751 (55.0) T2 (before closure): 366 (58.7) T2 (after school closure): 385 (36.1) Sensitivity analysis: 320	11–18; 14.82 (1.57); Adol	Psychosocial Sense of School Membership short version-self-report	Post-traumatic stress symptoms through Children’s Revised Impact of Events Scale-8—self-report	For subsample who completed T2 before school closures: T1: 42.02 (9.72) T2: 40.31 (10.99) For subsample who completed T2 after school closures: T1: 43.09 (9.73) T2: 42.75 (10.37) Min = 9 Max = 45 Mean levels changes of loneliness for students who completed before school closure vs. after school closure was not significant (i.e., change not due to school closure)	Change in trauma→change in school belonging: Overall Sample: *b* = −0.061, *se* = 0.057, *t* = −1.062, *p* = 0.288 Subsample for sensitivity analysis (*n* = 320): *b* = −0.429, *se* = 0.193, *t* = −2.221, *p* = 0.027 Change in trauma x COVID-19 school closure→Change in school belonging COVID: *b* = 0.472 *se* = 0.213, *t* = 2.219, *p* = 0.029 Summary: No impact of change in trauma by change in school belonging for overall sample. Sensitivity analyses on sample with mix of school closure showed as trauma symptoms increased, school belonging decreased only for students who completed T2 after school closure.	T1 and T2: 3–6 months apart T2: *n* = 336 before school closure T2: *n* = 386 after school closure (T2 was an intervention period that overlapped the pandemic for some students) Subsample for sensitivity analysis had mix of before and after closure	7
Westrupp et al. [68], Australia	Com	1082 (48)	0–18; 8.7 (5.2); Both	Single item on loneliness from the CoRonavIrus Health Impact Survey-parent-report	Short Mood and Feelings Questionnaire Adapted Brief Spence Children’s Anxiety Scale -parent-report	During baseline: Full Sample: 2.3 (1.0) Locked-down Victorian Sample: 2.3 (1.0) Non-Victorian Sample: 2.4 (1.0) Min = 1 Max = 5	Summary of loneliness→Dep and Anx: Baseline loneliness predicted elevated trajectories of Dep and Anx among locked-down Victorian sample and non-Victorian sample; Locked-down Victorian samples had peak in mental health symptoms compared to other areas.	T1: 8–28 April 2020 T2 onwards: 14 time points every 2–4 weeks until 19 May 2021; both school closure and reopening assessed	7
Zhu et al. [69], Hong Kong	Com	1491 (46.61)	10–17; 13.04 (0.86); Both	T2: single item on feeling lonely-self-report	T1 and T2: Suicide ideation-single item from Patient Health Questionnaire-9-self-report	0.51 (0.86) Min = 0 Max = 3	Suicide ideation (SI) comparing pre-COVID-19 to during COVID-19: -Non-SI: (65.0%) -Recovered SI: (14.0%) -Occurrent SI: (10.7%) -Recurrent SI: (10.4%) Summary of group mean differences in loneliness: Loneliness was highest in Occurrent and Recurrent groups compared to Non-SI and Recovered SI groups, but the former two groups were not different from one another	T1: Sept 2019 T2: June 2020, during schools reopened	5
Zuccolo et al. [70], Brazil	Com	5795 (50.77) Follow up: 3224 (51.27)	5–17; Baseline: 10.7 (3.63) Follow up: 10.6 (3.61); Both	Single item of child feeling lonely-parent-report	Revised Children’s Anxiety and Depression Scale-parent-report	Baseline group proportions: Never/almost never: 33.04% A few times: 53.10% Often: 13.86% Follow up group proportions: Never/almost never: 32.25% A few times: 52.96% Often: 14.79%	Summary of T1 Loneliness→T1 Dep/Anx: Baseline (T1) feeling lonely a few times or often significantly predicted higher Dep and Anx compared to never/almost never lonely (*p* < 0.001 for all) Summary of T1 Loneliness→T2 Dep/Anx Baseline (T1) feeling lonely a few times or often significantly predicted higher Dep and Anx compared to never/almost never lonely (*p* < 0.001 for all)	T1: June–November 2020 T2: Every 15 days until June 2021	6

Note. When multiple statistics were reported for results, either the main univariate/multivariate results are reported or a summary description of the main pattern of results is reported; T1 = Time 1; T2 = Time 2; T3 = Time 3; T4 = Time 4; NA = Not Applicable or Not Provided; Both (under sample type) = Both community and clinical sample; Com = Community sample; Adol = Adolescent sample; Child = Child sample; Both (under sample age) = Both child and adolescent sample; (+) = positive association; (-) = negative association; NDD = Neurodevelopmental Disorder; Min = Minimum score; Max = Maximum score; Dep = Depression Symptoms; Anx = Anxiety Symptoms; Internalizing = Internalizing Symptoms; Externalizing = Externalizing Symptoms; Qual = Quality Rating.

## Data Availability

Details of studies coded in the systematic review are presented in Table 1 and Table 2.

## Supplementary Materials

The following supporting information can be downloaded at: https://www.mdpi.com/article/10.3390/children10020279/s1, Figure S1: Quality Assessment Questions; Table S1. Sample Search Strategy.

## References

[1] UNESCO (2021). Global Monitoring of School Closures. https://en.unesco.org/covid19/educationresponse#durationschoolclosures.

[2] Harris J.R. (1995). Where is the child’s environment? A group socialization theory of development. Psychol. Rev..

[3] De Jong G.J., van Tilburg T., Dykstra P.A., Vangelisti A.L., Perlman D. (2006). Loneliness and Social Isolation. The Cambridge Handbook of Personal Relationships.

[4] Perlman D., Peplau L.A., Gilour R., Duck S. (1981). Toward a Social Psychology of Loneliness. Personal Relationships in Disorder.

[5] Baumeister R.F., Leary M.R. (1995). The need to belong: Desire for interpersonal attachments as a fundamental human motivation. Psychol. Bull..

[6] Hawkley L.C., Cacioppo J.T. (2010). Loneliness matters: A theoretical and empirical review of consequences and mechanisms. Ann. Behav. Med..

[7] Lempinen L., Junttila N., Sourander A. (2018). Loneliness and friendships among eight-year-old children: Time-trends over a 24-year period. J. Child Psychol. Psychiatry.

[8] Surkalim D.L., Luo M., Eres R., Gebel K., van Buskirk J., Bauman A., Ding D. (2022). The prevalence of loneliness across 113 countries: Systematic review and meta-analysis. BMJ.

[9] Twenge J.M., Haidt J., Blake A.B., McAllister C., Lemon H., Le Roy A. (2021). Worldwide increases in adolescent loneliness. J. Adolesc..

[10] Qualter P., Brown S.L., Munn P., Rotenberg K.J. (2010). Childhood loneliness as a predictor of adolescent depressive symptoms: An 8-year longitudinal study. Eur. Child Adolesc. Psychiatry.

[11] Danneel S., Geukens F., Maes M., Bastin M., Bijttebier P., Colpin H., Verschueren K., Goossens L. (2020). Loneliness, social anxiety symptoms, and depressive symptoms in adolescence: Longitudinal distinctiveness and correlated change. J. Youth Adolesc..

[12] Kessler R.C., Amminger G.P., Aguilar-Gaxiola S., Alonso J., Lee S., Ustun T.B. (2007). Age of onset of mental disorders: A review of recent literature. Curr. Opin. Psychiatry.

[13] Solmi M., Radua J., Olivola M., Croce E., Soardo L., Salazar de Pablo G., Shin J.I., Kirkbride J.B., Jones P., Kim J.H. (2022). Age at onset of mental disorders worldwide: Large-scale meta-analysis of 192 epidemiological studies. Mol. Psychiatry.

[14] Page M.J., McKenzie J.E., Bossuyt P.M., Boutron I., Hoffmann T.C., Mulrow C.D., Shamseer L., Tetzlaff J.M., Akl E.A., Brennan S.E. (2021). The PRISMA 2020 statement: An updated guideline for reporting systematic reviews. Syst. Rev..

[15] Ernst M., Niederer D., Werner A.M., Czaja S.J., Mikton C., Ong A.D., Rosen T., Brähler E., Beutel M.E. (2022). Loneliness before and during the COVID-19 pandemic: A systematic review with meta-analysis. Am. Psychol..

[16] Loades M.E., Chatburn E., Higson-Sweeney N., Reynolds S., Shafran R., Brigden A., Linney C., McManus M.N., Borwick C., Crawley E. (2020). Rapid systematic review: The impact of social isolation and loneliness on the mental health of children and adolescents in the context of COVID-19. J. Am. Acad. Child Adolesc. Psychiatry.

[17] Buecker S., Horstmann K.T. (2021). Loneliness and social isolation during the COVID-19 pandemic. Eur. Psychol..

[18] Kauhanen L., Wan Mohd Yunus W.M.A., Lempinen L., Peltonen K., Gyllenberg D., Mishina K., Gilbert S., Bastola K., Brown J.S.L., Sourander A. (2022). A systematic review of the mental health changes of children and young people before and during the COVID-19 pandemic. Eur. Child Adolesc. Psychiatry.

[19] Viner R., Russell S., Saulle R., Croker H., Stansfield C., Packer J., Nicholls D., Goddings A.L., Bonell C., Hudson L. (2022). School closures during social lockdown and mental health, health behaviors, and well-being among children and adolescents during the first COVID-19 wave: A systematic review. JAMA Pediatr..

[20] Ma L., Mazidi M., Li K., Li Y., Chen S., Kirwan R., Zhou H., Yan N., Rahman A., Wang W. (2021). Prevalence of mental health problems among children and adolescents during the COVID-19 pandemic: A systematic review and meta-analysis. J. Affect. Disord..

[21] Racine N., McArthur B.A., Cooke J.E., Eirich R., Zhu J., Madigan S. (2021). Global prevalence of depressive and anxiety symptoms in children and adolescents during COVID-19: A meta-analysis. JAMA Pediatr..

[22] Nearchou F., Flinn C., Niland R., Subramaniam S.S., Hennessy E. (2020). Exploring the impact of COVID-19 on mental health outcomes in children and adolescents: A systematic review. Int. J. Environ. Res. Public Health.

[23] Samji H., Wu J., Ladak A., Vossen C., Stewart E., Dove N., Long D., Snell G. (2022). Review: Mental health impacts of the COVID-19 pandemic on children and youth—A systematic review. Child Adolesc. Ment. Health.

[24] Chaabane S., Doraiswamy S., Chaabna K., Mamtani R., Cheema S. (2021). The impact of COVID-19 school closure on child and adolescent health: A rapid systematic review. Children.

[25] Theberath M., Bauer D., Chen W., Salinas M., Mohabbat A.B., Yang J., Chon T.Y., Bauer B.A., Wahner-Roedler D.L. (2022). Effects of COVID-19 pandemic on mental health of children and adolescents: A systematic review of survey studies. SAGE Open Med..

[26] Erzen E., Çikrikci Ö. (2018). The effect of loneliness on depression: A meta-analysis. Int. J. Soc. Psychiatry.

[27] Hards E., Loades M.E., Higson-Sweeney N., Shafran R., Serafimova T., Brigden A., Reynolds S., Crawley E., Chatburn E., Linney C. (2022). Loneliness and mental health in children and adolescents with pre-existing mental health problems: A rapid systematic review. Br. J. Clin. Psychol..

[28] (2022). Covidence Systematic Review Software, Veritas Health Innovation. www.covidence.org.

[29] Cauberghe V., Van Wesenbeeck I., De Jans S., Hudders L., Ponnet K. (2021). How adolescents use social media to cope with feelings of loneliness and anxiety during COVID-19 lockdown. Cyberpsychol. Behav. Soc. Netw..

[30] National Institutes of Health (2021). Quality Assessment Tool for Observational Cohort and Cross-Sectional Studies. https://www.nhlbi.nih.gov/health-topics/study-quality-assessment-tools.

[31] Boursier V., Gioia F., Musetti A., Schimmenti A. (2022). COVID-19-related fears, stress and depression in adolescents: The role of loneliness and relational closeness to online friends. J. Hum. Behav. Soc. Environ..

[32] Dondi A., Fetta A., Lenzi J., Morigi F., Candela E., Rocca A., Cordelli D., Lanari M. (2021). Sleep disorders reveal distress among children and adolescents during the Covid-19 first wave: Results of a large web-based Italian survey. Ital. J. Pediatr..

[33] Dubois-Comtois K., Suffren S., St-Laurent D., Milot T., Lemelin J.P. (2021). Child psychological functioning during the COVID-19 lockdown: An ecological, family-centered approach. J. Dev. Behav. Pediatr..

[34] Ellis W.E., Dumas T.M., Forbes L.M. (2020). Physically isolated but socially connected: Psychological adjustment and stress among adolescents during the initial COVID-19 crisis. Can. J. Behav. Sci. Rev. Can. Des Sci. Du Comport..

[35] Espinoza G., Hernandez H.L. (2022). Adolescent loneliness, stress and depressive symptoms during the COVID-19 pandemic: The protective role of friends. Infant Child Dev..

[36] Fernandes M.D.S.V., Silva T.M., Noll P.R.E.S., Almeida A.A.D., Noll M. (2022). Depressive symptoms and their associated factors in vocational–technical school students during the COVID-19 pandemic. Int. J. Environ. Res. Public Health.

[37] Fogarty A., Brown S., Gartland D., Mensah F., Seymour M., Savopoulos P., FitzPatrick K., Papadopoullos S., Giallo R. (2022). Psychosocial factors associated with adolescent depressive and anxiety symptoms during the COVID-19 pandemic. Int. J. Behav. Dev..

[38] Gilsbach S., Herpertz-Dahlmann B., Konrad K. (2021). Psychological impact of the COVID-19 pandemic on children and adolescents with and without mental disorders. Front. Public Health.

[39] Hou T., Xie Y., Mao X., Liu Y., Zhang J., Wen J., Chen Y., Luo Z., Cai W. (2021). The mediating role of loneliness between social support and depressive symptoms among Chinese rural adolescents during COVID-19 uutbreak: A comparative study between left-behind and non-left-behind students. Front. Psychiatry.

[40] Jones S.E., Ethier K.A., Hertz M., DeGue S., Le V.D., Thornton J., Lim C., Dittus P.J., Geda S. (2022). Mental health, suicidality, and connectedness among high school students during the COVID-19 pandemic—Adolescent Behaviors and Experiences Survey, United States, January–June 2021. MMWR Suppl..

[41] Kayaoğlu K., Başcıllar M. (2022). Determining the relationship between loneliness and depression in adolescents during the COVID-19 pandemic: A cross-sectional survey. J. Child Adolesc. Psychiatr. Nurs..

[42] Kilinçel S., Muratdagi G. (2021). Evaluation of factors affecting social media addiction in adolescents during the COVID-19 pandemic. Ann. Clin. Anal. Med..

[43] Laslo-Roth R., George-Levi S., Rosenstreich E. (2021). Protecting children with ADHD against loneliness: Familial and individual factors predicting perceived child’s loneliness. Personal. Individ. Differ..

[44] Li S.H., Beames J.R., Newby J.M., Maston K., Christensen H., Werner-Seidler A. (2022). The impact of COVID-19 on the lives and mental health of Australian adolescents. Eur. Child Adolesc. Psychiatry.

[45] Li J., Zhan D., Zhou Y., Gao X. (2021). Loneliness and problematic mobile phone use among adolescents during the COVID-19 pandemic: The roles of escape motivation and self-control. Addict. Behav..

[46] Liu J., Zhou T., Yuan M., Ren H., Bian X., Coplan R.J. (2021). Daily routines, parent-child conflict, and psychological maladjustment among Chinese children and adolescents during the COVID-19 pandemic. J. Fam. Psychol..

[47] Low N., Mounts N.S. (2021). Economic stress, parenting, and adolescents’ adjustment during the COVID-19 pandemic. Fam. Relat..

[48] Murata S., Rezeppa T., Thoma B., Marengo L., Krancevich K., Chiyka E., Hayes B., Goodfriend E., Deal M., Zhong Y. (2021). The psychiatric sequelae of the COVID-19 pandemic in adolescents, adults, and health care workers. Depress. Anxiety.

[49] Oosterhoff B., Palmer C.A., Wilson J., Shook N. (2020). Adolescents’ motivations to engage in social distancing during the COVID-19 pandemic: Associations with mental and social health. J. Adolesc. Health.

[50] Palmer C.A., Oosterhoff B., Massey A., Bawden H. (2022). Daily associations between adolescent sleep and socioemotional experiences during an ongoing stressor. J. Adolesc. Health.

[51] Pan Y., Yang Z., Han X., Qi S. (2021). Family functioning and mental health among secondary vocational students during the COVID-19 epidemic: A moderated mediation model. Personal. Individ. Differ..

[52] Pfetsch J.S., Schultze-Krumbholz A., Lietz K. (2021). Can acting out online improve adolescents’ well-being during contact restrictions? A first insight into the dysfunctional role of cyberbullying and the need to belong in well-being during COVID-19 pandemic-related contact restrictions. Front. Psychol..

[53] Sette S., Zava F., Baumgartner E., Laghi F., Coplan R.J. (2022). Examining links between social withdrawal subtypes and internalizing problems among Italian primary school children. Eur. J. Dev. Psychol..

[54] Zuffiano A., Lopez-Perez B., McCagh J., Caprara G.V., Coplan R.J. (2022). Links between child shyness and indices of internalizing problems during the COVID-19 pandemic: The Protective role of positivity. J. Genet. Psychol..

[55] Soneson E., Puntis S., Chapman N., Mansfield K.L., Jones P.B., Fazel M. (2022). Happier during lockdown: A descriptive analysis of self-reported wellbeing in 17,000 UK school students during Covid-19 lockdown. Eur. Child Adolesc. Psychiatry.

[56] Varela J.J., Hernández C., Miranda R., Barlett C.P., Rodriguez-Rivas M.E. (2022). Victims of Cyberbullying: Feeling Loneliness and Depression among Youth and Adult Chileans during the Pandemic. Int. J. Environ. Res. Public Health.

[57] Wang J., Yang Y., Lin H., Richards M., Yang S., Liang H., Chen X., Fu C. (2021). Impact of psychosocial stressors on emotional and behavioral problems in Chinese adolescents during the COVID-19 period: The explanatory value of loneliness. Transl. Pediatr..

[58] Yilmaz M.B., Orhan F., Zeren S.G. (2022). Adolescent emotion scale for online lessons: A study from Turkey. Educ. Inf. Technol..

[59] Zhu S., Zhuang Y., Lee P., Li J.C., Wong P.W.C. (2021). Leisure and problem gaming behaviors among children and adolescents during school closures caused by COVID-19 in Hong Kong: Quantitative cross-sectional survey study. JMIR Serious Games.

[60] Alt P., Reim J., Walper S. (2021). Fall from grace: Increased loneliness and depressiveness among extraverted youth during the German COVID-19 lockdown. J. Res. Adolesc..

[61] Cooper K., Hards E., Moltrecht B., Reynolds S., Shum A., McElroy E., Loades M. (2021). Loneliness, social relationships, and mental health in adolescents during the COVID-19 pandemic. J. Affect. Disord..

[62] Houghton S., Kyron M., Hunter S.C., Lawrence D., Hattie J., Carroll A., Zadow C. (2022). Adolescents’ longitudinal trajectories of mental health and loneliness: The impact of COVID-19 school closures. J. Adolesc..

[63] Houghton S., Kyron M., Lawrence D., Hunter S.C., Hattie J., Carroll A., Zadow C., Chen W. (2022). Longitudinal trajectories of mental health and loneliness for Australian adolescents with-or-without neurodevelopmental disorders: The impact of COVID-19 school lockdowns. J. Child Psychol. Psychiatry.

[64] Magson N.R., Freeman J.Y.A., Rapee R.M., Richardson C.E., Oar E.L., Fardouly J. (2021). Risk and protective factors for prospective changes in adolescent mental health during the COVID-19 pandemic. J. Youth Adolesc..

[65] Rogers A.A., Ha T., Ockey S. (2021). Adolescents’ perceived socio-emotional impact of COVID-19 and implications for mental health: Results from a U.S.-based mixed-methods study. J. Adolesc. Health.

[66] Schwartz-Mette R.A., Duell N., Lawrence H.R., Balkind E.G. (2022). COVID-19 distress impacts adolescents’ depressive symptoms, NSSI, and suicide risk in the rural, northeast US. J. Clin. Child Adolesc. Psychol..

[67] Szelei N., Devlieger I., Verelst A., Spaas C., Jervelund S.S., Primdahl N.L., Skovdal M., Opaas M., Durbeej N., Osman F. (2022). Migrant students’ sense of belonging and the COVID-19 pandemic: Implications for educational inclusion. Soc. Incl..

[68] Westrupp E.M., Greenwood C.J., Fuller-Tyszkiewicz M., Olsson C.A., Sciberras E., Mikocka-Walus A., Melvin G.A., Evans S., Stokes M.A., Wood A.G. (2021). Parent and child mental health trajectories April 2020 to May 2021: Strict lockdown versus no lockdown in Australia. Aust. N. Z. J. Psychiatry.

[69] Zhu S., Zhuang Y., Lee P., Wong P.W.C. (2021). The changes of suicidal ideation status among young people in Hong Kong during COVID-19: A longitudinal survey. J. Affect. Disord..

[70] Zuccolo P.F., Casella C.B., Fatori D., Shephard E., Sugaya L., Gurgel W., Farhat L.C., Argeu A., Teixeira M., Otoch L. (2022). Children and adolescents’ emotional problems during the COVID-19 pandemic in Brazil. Eur. Child Adolesc. Psychiatry.

[71] Dunn C., Sicouri G. (2022). The relationship between loneliness and depressive symptoms in children and adolescents: A meta-analysis. Behav. Change.

[72] Eccles A.M., Qualter P. (2021). Review: Alleviating loneliness in young people—A meta-analysis of interventions. Child Adolesc. Ment. Health.

[73] Taylor R.D., Oberle E., Durlak J.A., Weissberg R.P. (2017). Promoting positive youth development through school-based social and emotional learning interventions: A meta-analysis of follow-up effects. Child Dev..

[74] Alegría M., Baum R., McCabe M.A., Williams J.L. (2021). School-Based Strategies for Addressing the Mental Health and Well-Being of Youth in the Wake of COVID-19.

[75] Vaillancourt T., McDougall P., Comeau J., Finn C., Blais J.M. (2021). COVID-19 school closures and social isolation in children and youth: Prioritizing relationships in education. Facets.

[76] Vaillancourt T. (2021). Physical Activity—The Forgotten Core Area of Child Development during the Pandemic. The Globe and Mail. https://www.theglobeandmail.com/canada/article-physical-activity-the-forgotten-core-area-of-child-development-during/.

[77] McGuine T., Biese K., Hetzel S., Schwarz A., Reardon C., Bell D., Brooks A., Dickman J., Watson A. (2022). A multi-year assessment of sport participation during the COVID-19 pandemic on the health of adolescent athletes. J. Athl. Train..

[78] Exner-Cortens D., Gaias L., Splett J.W., Jones J., Walker W. (2022). Embedding equity into school mental health theory, research, and practice: An introduction to the special issue series. Psychol. Sch..

[79] Wong J., Yi P.X., Quek F.Y.X., Lua V.Y.Q., Majeed N.M., Hartanto A. (2022). A four-level meta-analytic review of the relationship between social media and well-being: A fresh perspective in the context of COVID-19. Curr. Psychol..

