# Supplementary material for: Loneliness and Well-Being in Children and Adolescents during the COVID-19 Pandemic: A Systematic Review
Source: Children (Basel). 2023 Jan 31;10(2):279. doi: 10.3390/children10020279 (PMC9955087; doi:10.3390/children10020279)
Supplement: Supplementary file 1 [file children-10-00279-s001.zip › children-2125756-supplementary.pdf]

Supplemental Materials

Adapted from the NIH Quality Assessment Tool for Observational Cohort and Cross-Sectional Studies to evaluate any bias in the study or measurements. This measure is available at https://www.nhlbi.nih.gov/health-topics/study-quality-assessment-tools

Rated as 0 = No or 1 = Yes. If not applicable or unclear, coded as 0 = No.

1. Was the research question or objective clearly stated?
2. Was the study population clearly specified and defined?
3. Was the participation rate of eligible persons at least 50%?
4. Was the sample size justification, power description, or variance and effect estimates provided?
5. Were the measures of loneliness clearly defined, valid, and reliable?
6. Were the measures of well-being/ mental health clearly defined, valid, and reliable?
7. Was the study longitudinal in design (more than one assessment)?
8. Were the analyses appropriate for the research objective (i.e., specifically answers research question)?
9. Were key potential confounding variables measured and adjusted statistically for their impact?
10. Was the loss to follow-up after base line 20% or less (*longitudinal studies only*)?

Figure S1. Quality Assessment Questions.

Table S1. Sample Search Strategy.

#	Searches
1	exp COVID-19/
2	(COVID-19 or COVID19 or coronavirus or corona virus).ab,ti.
3	exp Loneliness/
4	exp Social Isolation/
5	(lonely or loneliness or "social isolation" or "belonging" or "mattering").ab,ti.
6	exp mental health/ or exp mental disorders/ or exp anxiety disorders/ or exp mood disorders/ or exp substance-related disorders/
7	(well-being or "well being" or wellbeing or "mental health" or "mental disorder*" or "mental illness").ab,ti.
8	("internalizing difficulties" or "internalizing symptoms" or "internalizing problems" or "internalizing challenges").ab,ti.
9	("externalizing difficulties" or "externalizing symptoms" or "externalizing problems" or "externalizing challenges").ab,ti.
10	1 or 2
11	3 or 4 or 5
12	exp Anxiety/
13	exp Depression/
14	(anxiety or anxious* or depress*).ab,ti.
15	exp alcohol-related disorders/ or exp marijuana abuse/

16	("alcohol use" or "cannabis use" or "marijuana use" or "alcohol consumption" or "cannabis consumption" or "marijuana consumption" or "alcohol abuse" or "cannabis abuse" or "marijuana abuse" or "substance use" or "substance abuse" or "substance misuse").ab,ti.
17	Adolescent/
18	Child/
19	(Child* or adolescen* or youth* or "elementary school" or "elementary student*" or "middle school" or "high school" or "high school student*" or "middle school student" or "secondary school" or "secondary student" or teen*).ab,ti.
20	17 or 18 or 19
21	6 or 7 or 8 or 12 or 13 or 14 or 15 or 16
22	10 and 11 and 20 and 21
23	limit 22 to yr="2020 - 2022"
